# Dietary Trends and Lifestyle Habits Among University Students: Analysis of Consumption Patterns and Nutritional Risks

**DOI:** 10.3390/nu18030532

**Published:** 2026-02-05

**Authors:** Alejandra Vázquez-Aguilar, Juan Manuel Ballesteros-Torres, Anayansi Escalante-Aburto, César Huerta-Canseco, Karla Lizbet Jiménez-López, Cindy Joanna Caballero-Prado

**Affiliations:** 1 Department of Health Sciences, University Center of the Valleys, Universidad de Guadalajara, Carr. a Guadalajara km. 45.5, Ameca 46600, Jalisco, Mexico; alejandra.vazquez@academicos.udg.mx; 2 Departamento de Microbiología e Inmunología, Facultad de Ciencias Biológicas (FCB), Universidad Autónoma de Nuevo León (UANL), Ave. Universidad s/n, Cd. Universitaria, San Nicolás de los Garza 66450, Nuevo León, Mexico; juan.ballesterostr@uanl.edu.mx; 3 Tecnologico de Monterrey, Institute for Obesity Research, Ave. Eugenio Garza Sada 2501, Monterrey 64849, Nuevo León, Mexico; 4 Tecnologico de Monterrey, School of Engineering and Sciences, Ave. Eugenio Garza Sada 2501, Monterrey 64849, Nuevo León, Mexico; 5 Departamento de Nutrición, Escuela de Ciencias Aliadas de la Salud, Universidad de Monterrey, Av. Morones Prieto 4500 Pte., San Pedro Garza García 66238, Nuevo León, Mexico; cesar.huertac@udem.edu; 6 Department of Social Sciences, Southern University Center, University of Guadalajara, Ciudad Guzmán 49000, Jalisco, Mexico; lizbet.jimenez@cusur.udg.mx

**Keywords:** dietary patterns, food consumption, students population, nutrition, healthy foods, nutritional risk

## Abstract

**Background/Objectives**: The global prevalence of overweight and obesity among young adults has doubled since 1975, primarily due to unhealthy dietary habits and sedentary lifestyles. Understanding dietary patterns (DPs) in this population is essential for designing effective prevention strategies. This study aimed to characterize the dietary patterns and diet quality of university students and to examine their physical activity and associated health risks. **Methods**: A convenience sample of 136 participants (77.9% females, 22.1% males) was recruited. Data on clinical history, lifestyle behaviors, and physical activity were collected using a structured questionnaire. Dietary intake was assessed using a food frequency questionnaire and three 24-h dietary recalls. Intake was analyzed by food groups, total energy, and macronutrient and fiber composition. Principal component analysis was applied to identify DPs. **Results**: Three major DPs were identified: Ultra-Processed Foods, Variety Foods, and Traditional Mixed Mexican. Overall, participants showed low consumption of fiber, legumes, and nuts, coupled with high intake of animal-based foods. The mean daily energy intake was 2278 kcal for men and 2008 kcal for women. Although participants demonstrated higher adherence to the Traditional Mixed Mexican pattern, a strong tendency toward the Ultra-Processed Foods pattern was observed, which is linked to an increased risk of chronic diseases and poor nutritional outcomes. **Conclusions**: The findings highlight the urgent need for targeted dietary interventions among university students. Strategies should emphasize increased intake of fiber-rich plant foods, moderation of protein consumption, and reduction in refined carbohydrates and added sugars to promote healthier dietary habits and prevent chronic disease development.

## 1. Introduction

Diet plays a fundamental role in individual health and well-being, particularly during the university stage, which is often marked by significant lifestyle changes and increased autonomy in food choices, often leading to unhealthy choices [1]. This stage is a crucial point, as students undergo changes in their lifestyles that affect their food habits and preferences [2]. This transition often leads to poorer dietary patterns, as individuals have less time to purchase fresh ingredients or prepare meals, prompting them to choose processed foods that are faster and more convenient to prepare [3]. Some studies have reported low diet quality among young students, characterized by a high consumption of unhealthy foods, with a high contribution of fats and sugars, and a low intake of fruits and vegetables [4,5,6].

Overweight and obesity are associated with multiple cardiometabolic diseases and have a negative impact on health [7]. In Mexico, the prevalence of overweight and obesity in young people over 20 years of age was 38.3%, where 41.2% were men and 35.8% were women. Obesity was reported at a rate of 36.9%, with 41.0% of cases affecting women and 32.3% affecting men [8]. According to Ponce et al. [9], the prevalence of overweight and obesity in young people has doubled since 1975 due to changes in dietary patterns and lack of physical activity. In addition to these risk factors, there is the tobacco habit, with a consumption rate of 4.6% among adolescents and 19.5% among adults over 20 years of age. This means that approximately 1 million adolescents and 16.6 million adults are tobacco users [10]. Similarly, alcohol is a factor that aggravates the problems described above. According to Ramírez-Toscano et al. [11], the prevalence of current alcohol consumption in the Mexican adult population was 55.5%, being significantly higher in men with 67.3% than in women with 44.6%. This study aims to assess the dietary patterns of university students and examine their lifestyle behaviors, including physical activity levels, to identify prevailing consumption trends and potential nutritional risks among this population.

## 2. Materials and Methods

### 2.1. Study Design and Volunteers

The present study employed an observational, descriptive, cross-sectional design and was conducted in accordance with the principles of the Declaration of Helsinki. The study protocol was reviewed and approved by the Institutional Research Committee of the Universidad de Monterrey (protocol code 17092024-NUT-EM-CI) on 17 September 2024. Participants were male and female university students aged 18 to 25 years who voluntarily agreed to participate and provided written informed consent. Exclusion criteria included adherence to a controlled or restrictive diet, use of dietary supplements, presence of food allergies or intolerances, diagnosis of chronic degenerative diseases, and adherence to restrictive dietary patterns, such as vegetarian or ketogenic diets.

A range of communication channels was employed to disseminate information about the study and recruit participants from the university student population. Recruitment strategies included in-person classroom visits, during which students were invited to participate, as well as remote outreach via the university’s official communication platforms, primarily its institutional social media channels. Additionally, printed informational flyers were distributed across campus.

Following recruitment, students who met the inclusion criteria and provided written informed consent were enrolled. A convenience sample of 136 participants was obtained, consisting of 77.9% females and 22.1% males. The Ethics and Research Committee approved the methodology used in this study.

### 2.2. Sociodemographic, Clinical, and Anthropometric Data Assessment

Before enrollment, participants provided written informed consent in compliance with the Mexican Official Standard NOM-012-SSA3-2012 [12], having received comprehensive information regarding the study objectives and approved the use and publication of their data prior to questionnaire administration [12]. Subsequently, each participant was called to a first appointment (in person) to apply the different questionnaires for data collection (digital clinical history), which included sociodemographic data, and anthropometric data, such as weight (kg) and height (cm) to obtain the Body Mass Index (BMI, kg/m^2^), lifestyle habits, physical activity smoking habits, alcohol consumption habits. Also, a food consumption questionnaire (FCQ) and three 24-h dietary recalls (one conducted during the interview and two on different days of the week) were applied. During the face-to-face interview, participants were informed that they would be required to complete two additional 24-h dietary recalls on non-consecutive days. To facilitate data collection, a Google Forms^®^ questionnaire was subsequently provided via a secure link for submitting dietary information. Participants were weighed and measured according to the procedures recommended by the World Health Organization (WHO) [13]. An OMRON HBF-222T electronic scale (Yangzhou Jiangsu, China) was used to measure body weight, and a Seca 213 stadiometer was used to measure height. BMI was calculated using the formula weight (kg) divided by height (m^2^) and was classified according to the WHO criteria [14]. To evaluate physical activity, we used a validated questionnaire that asked participants about the frequency of their physical exercise, sports, and other physical activities, measured in days per week. It also inquired about the duration of these activities in hours and minutes [15].

### 2.3. Dietary Pattern Analysis

A semi-quantitative tool assessing 34 food items was used to evaluate food intake, with foods included in the 2021 National Health and Nutrition Survey for adolescents and adults aged 12 years and older [16]. Participants were asked to report the frequency and quantity of consumption (i.e., number of times per specified period) for each food item. Responses were recorded using a Likert-type scale, ranging from “never or rarely” to “six or more times per day.” These responses were subsequently converted to quantitative values, enabling the estimation of daily food intake (g/day) for each item. Subsequently, using this information about foods and beverages, they were classified into 20 food groups based on their nutritional content using the Mexican Food and Food Equivalents System as a reference [17]. These groups were used to perform principal component analysis (PCA) and identify the foods most consumed by the study participants.

Additionally, three 24-h dietary recalls were conducted on different days of the week, to ensure adequate collection of dietary data in the 24-h recall, a form was designed that included all food groups included in the Mexican Food and Food Equivalents System [MFFES] [17] in portions, at each meal time, in 24 h, taking into account foods consumed at breakfast, morning snack, lunch, afternoon snack, and dinner. With this instrument, participants had five response options regarding the number of portions they consumed at each mealtime: (a) zero portions, (b) half portion, (c) 1–2 portions, (d) 3–4 portions, and (e) 5 or more portions.

Based on the collected data, the frequency of consumption and intake of each food item was calculated and subsequently categorized into the following groups: vegetables, fruits, legumes, dairy products, white meat, red meat, oils and fats, sugar-sweetened alcoholic beverages, confectionery and sweets, fried foods, sugar-sweetened beverages, and foods with added sugars. Each item reported in the 24-h dietary recalls was quantified in terms of energy intake and grams per day (g/day) [17]. Afterwards, all foods and beverages were classified according to their nutritional composition and similarity, yielding a total of nine food groups for analysis.

### 2.4. Data Processing and Statistical Analysis

Once all questionnaires were completed, a database was created to analyze sociodemographic, anthropometric, clinical, and dietary variables, and to estimate daily (24-h) energy intake, macronutrient consumption, and food group intake. After data review and cleaning, a small proportion of missing data (<10%) was identified and treated as missing values. Additionally, 19 participants with atypical values or incomplete records were excluded from the analysis.

Descriptive statistics were calculated and are presented as frequencies and percentages, with participants categorized according to self-reported gender. The normality of continuous variables was assessed using the Kolmogorov–Smirnov test. For variables with a normal distribution, group differences were evaluated using Student’s *t*-test, whereas the Mann–Whitney U test was applied for variables with non-normal distributions. A two-tailed significance level of *p* ≤ 0.05 was considered statistically significant for all analyses. Statistical analyses were performed using SPSS Statistics software, version 22.0. Multivariate PCA was employed to identify DPs, and Varimax rotation was applied to maintain non-correlation and enhance precision and interpretability. Data feasibility was confirmed using the Kaiser-Meyer-Olkin sampling adequacy test (KMO = 0.60). Although this value is considered marginal according to conventional criteria, it is generally regarded as acceptable within the context of nutritional epidemiology, and Bartlett’s test of sphericity (*p* < 0.001) indicated that the sample’s statistical values were reliable. The number of principal components was determined using a graphical representation of the extracted components based on their eigenvalues. Factor loadings, which are the coefficients representing each variable’s contribution to the derived patterns (DPs), were considered significant when their absolute values were greater than or equal to 0.30. This threshold was chosen because it provided a better fit to the data [18].

## 3. Results

In this study, a total of 136 young university students participated; the female gender represented 77.9% and the male gender 22.1%, 52% practiced physical activity regularly, 36% reported having the habit of smoking, 61% reported drinking alcohol on weekends, and a BMI was observed within the normal range for both women and men, 22.32 and 22.41 kg/m^2^ for woman and men, respectively (Table 1).

The consumption of vegetables and fruits was found to be adequate, with an average intake of 4 portions of vegetables and 3 portions of fruits per day. However, the intake of legumes was low, averaging 1.5 portions, and nuts were consumed even less frequently, at less than 1 portion per day. The average intake of cereals and grains was adequate. In contrast, foods of animal origin, particularly meat and dairy products, were consumed in excess, especially among men compared to women. A similar trend was observed for the consumption of sugar-sweetened beverages and sugary foods (see Table 2).

The analysis of the diet based on caloric energy and macronutrients (Table 3) revealed that caloric intake was ~2278 kcal for men and ~2008 kcal for women, indicating adequate intake for their age and daily activities. In terms of macronutrients, carbohydrate consumption was 56% and 58%, respectively, for men and women, which aligns with the recommendations for a healthy diet of 2000 calories [20]. In terms of protein consumption, men showed a contribution of 39%, while women had a contribution of 37%. This indicates that their intake nearly doubled the recommended amount. Lipids contributed 21% and 20%, indicating an adequate intake of lipids in the diet. Regarding fiber, a relatively low intake was observed at 19 g/day in both groups, indicating that neither group met the recommendation of at least 30 g/day.

**Figure 1:**
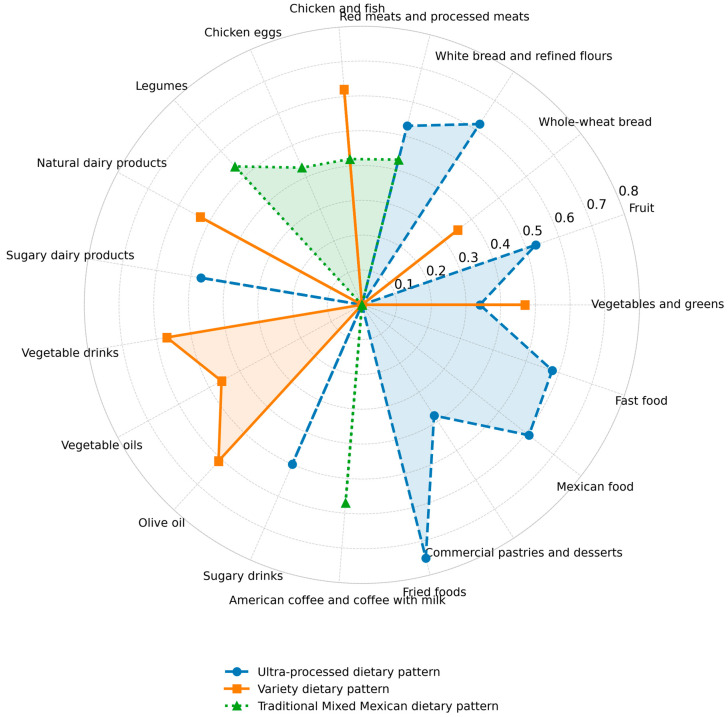
Factor loading matrix of dietary patterns identified in the university students.

Figure 1 shows the food groups included in each of the three dietary patterns (DPs) identified in our study, considering a score of ≥0.30 as significant in the factor-loading matrix for the food groups that comprise each DP (data shown in Supplementary Table S1). The observed patterns explained 35.89% of the total variance in food consumption among the study participants. The first category, “Ultra-processed DPs”, was defined by the consumption of white bread and refined flours, sweet bread, red meats and processed meats, processed and dairy products with added sugar, vegetable oils (soybean, corn, and canola), beverages with added sugar, fried foods, commercial pastries and desserts, and fast food. The second category, referred to as “Variety-Food DPs”, included the consumption of vegetables and green fruits, whole-wheat bread, sweet bread, chicken and fish, natural dairy products, plant-based beverages (such as almond and soy), olive oil, commercial pastries, and desserts. Lastly, the third category, known as “Traditional Mixed Mexican DPs,” included a variety of foods such as vegetables, greens, fruits, red meats, processed meats, chicken, fish, legumes, eggs, and coffee with milk.

Table 4 shows the percentage of adherence by gender for the various identified dietary patterns (DPs). The Traditional Mixed Mexican DP had the highest adherence rate at 37%, followed closely by the ultra-processed DP at 34% and the variety DP at 28.9%. This indicates a fairly similar distribution of dietary patterns across the study population.

## 4. Discussion

The current study assessed the eating habits and lifestyles of 136 university students aged 18 to 25 years. The main finding was the identification of three dietary patterns that highlight the most common consumption trends in this group of students (Figure 1). A dietary pattern called “ultra-processed” was identified, which was characterized by the consumption of white bread and refined flours, sweet bread, red and processed meats, processed and dairy products, vegetable oils (soy, corn, and canola), sugary drinks, fried foods, commercial pastries and desserts, and fast food. The second dietary pattern, “variety of foods”, was characterized by the consumption of green vegetables and fruits, whole-wheat bread, sweet bread, chicken and fish, natural dairy products, plant-based beverages (almond and soy), olive oil, commercial pastries, and desserts. The third dietary pattern was named “Traditional Mixed Mexican” and included the consumption of vegetables, fruits, red and processed meats, chicken, fish, legumes, eggs, and coffee with milk. Additionally, lifestyle behaviors identified as risk factors included tobacco use in 15.4% of university students and alcohol consumption during weekends reported by 61.8% of participants.

These findings are consistent with previous studies reporting comparable DPs [2,21,22,23], indicating that diverse eating behaviors are observed across different regions worldwide. Such patterns commonly include Westernized and healthy or cautious DPs [22], as well as traditional patterns characterized by the consumption of locally sourced and culturally specific foods [23]. Conversely, DPs marked by a high intake of alcohol, convenience foods, red and processed meats have also been frequently identified [2].

Notably, although the Traditional Mixed Mexican DP showed the highest adherence (37%) in this group, the Ultra-processed DPs also exhibited a high level of adherence (34%) (Table 4). This finding agrees with the study by Betancourt-Núñez et al. [24], who reported that adherence to a Westernized dietary pattern is positively associated with being under 22 years of age. The Westernized pattern is broadly defined by a high consumption of ultra-processed foods, commercially prepared products, red meat, and sugar-sweetened beverages [25]. This DP has been associated with an increased risk of cardiovascular disease (CVD) and metabolic disorders [26]. In Mexico, ultra-processed foods make up about 30% of total energy intake, leading to decreased dietary diversity and lower nutrient density, as well as a higher risk of chronic non-communicable diseases [9,27].

Although participants’ BMI was within the normal range, 44.8% reported low physical activity levels or sedentary behavior. This finding is concerning, as the persistence of such lifestyle behaviors may contribute to the development of “normal-weight obesity”, a condition characterized by normal BMI but elevated body fat percentage and increased metabolic dysregulation, which is associated with a higher risk of metabolic syndrome and cardiometabolic dysfunction [28]. Likewise, comparable BMI values (~19–22 kg/m^2^) have been reported among university students consuming protein supplements in controlled diets and moderate physical activity [29].

Analysis of daily food group portion consumption among university students revealed gender differences, particularly in the intake of animal-based foods and sugar-sweetened beverages, with men consuming more than women (Table 2). The average intake for both genders exceeded the WHO-recommended range [20]. These patterns may have potential long-term health implications. While animal-source proteins are essential for growth and maintenance, excessive consumption, particularly when unbalanced by fiber and plant-based foods, can contribute to increased acidic load, lipotoxicity, and an inflammatory milieu in metabolically vulnerable individuals.

On the other hand, fruit and vegetable intake was generally adequate in both genders and consistent with Mexican dietary guidelines [30]. However, the total dietary fiber intake remained below recommended levels, suggesting limitations in the quality, variety, or portion size of the plant-based foods consumed, including legumes and nuts. This insufficient fiber intake (19 g/day) constitutes a significant risk factor, given the well-established role of fiber in cardiometabolic health. Moreover, limited consumption of these foods (high fiber and healthy fats) implies reduced intake of beneficial lipids and a loss of the protective cardiometabolic effects associated with regular nut consumption, which is particularly concerning among young adults undergoing critical developmental and behavioral transitions.

Consuming more than eight servings of cereal and grain per day likely indicates a preference for refined grains, which aligns with Ultra-processed dietary patterns (Figure 1). In this sense, high intake of refined carbohydrates is associated with insulin resistance, glycemic variability, and central adiposity. Pereira-Santos et al. [31] found that Brazilian students have diets high in simple carbohydrates and saturated fats, while lacking fiber and essential nutrients. This pattern reflects concerns across various cultures, and the similarity suggests a global concern about university students’ dietary choices.

The increased consumption of sugar-sweetened beverages, especially among men, exacerbates these risks. Although consumption remained within upper dietary limits, habitual intake of added sugars is associated with low-grade systemic inflammation and metabolic dysregulation in young adults. The transition to university life often leads to increased consumption of processed foods, sugary snacks, and alcohol. Evidence suggests that students frequently struggle to maintain a balanced diet due to time, financial, and social constraints [32]. Additionally, cooking skills significantly influence food choices, suggesting that students with better cooking skills tend to adhere to a healthier diet [33]. These findings indicate a shift toward a Westernized DPs among university students, which may increase the risk of chronic non-communicable diseases despite normal BMI (“normal-weight obesity”). Evidence shows that adherence to healthy dietary patterns, such as the Mediterranean diet, is associated with better mental health, lower stress, and improved academic performance [34]. In contrast, unhealthy diets are linked to obesity, mental health disorders, and poor sleep quality [35,36].

Regarding dietary energy intake, our results show that participants had adequate intake, based on the WHO dietary recommendations for adults aged 18–60 years, which propose an average reference intake of 2000 kcal/day [20]. Carbohydrate intake also complies with WHO recommendations, both in absolute terms (225–335 g/day) and relative terms (45–65% of total caloric intake).

In both genders, lipid intake was at the lower limit of the WHO-recommended range (20–35% of total energy intake), a pattern previously observed in young populations [37]. This may reflect sociocultural influences, including body image concerns and the persistent perception that healthy diets should restrict fat intake [38,39]. However, adequate intake of lipids—particularly polyunsaturated and monounsaturated fatty acids—is essential for long-term health. Evidence suggests that low-fat intake may adversely affect hormonal balance [40] and has been associated with menstrual disorders and reduced bone health in adult women [41]. The relevance of health awareness in this population has also been highlighted in studies reporting elevated cardiometabolic risk factors among university students [42].

On the other hand, protein intake was found to be considerably higher than WHO recommendations for this population group (10–15% of total caloric intake). In this study group, protein intake accounted for 39% in men and 37% in women, exceeding the recommended level by more than twice. This finding is consistent with data from other young populations following Western dietary patterns and with findings in individuals practicing strength or weightlifting [43,44]. While a moderately high protein intake (1.5–2.0 g/kg of body weight, which is 10–30% of total caloric intake) has been linked to benefits for muscle mass and glycemic control, it does not negatively impact kidney function in healthy individuals [45]. Chronic high-protein intake (above 25% of total calories) elicits complex metabolic responses, including the release of peptide hormones from the gastrointestinal tract and increased concentrations of circulating amino acids and their metabolites [45].

Research suggests that an excess of amino acids, coupled with low intake of fruits and vegetables and an imbalance in macrophage activity, may contribute to low-grade chronic inflammation associated with obesity. This combination could increase the risk of cardiovascular disease, disrupt bone metabolism, and affect acid-base balance [46].

These findings highlight the importance of monitoring the early adoption of inadequate DPs, such as hyperproteic diets and high intake of simple or refined sugars, particularly during early adulthood. Other habits, such as smoking, high consumption of ultra-processed foods, and low intake of fruits, vegetables, and fiber, tend to persist over time, contributing to the development of low-grade chronic inflammation, increased adipose tissue, and, consequently, a higher cardiovascular risk, one of the leading causes of death worldwide [47,48].

## 5. Conclusions

This study identified three DPs among Mexican university students, with higher adherence to a Traditional Mixed Mexican DPs alongside a notable tendency toward an Ultra-processed DPs, which raises concern due to its association with poor nutritional quality and chronic disease risk. Participants met the WHO recommendations for total energy intake, while their consumption of fruits, vegetables, and protein exceeded the recommended levels. However, the intake of legumes, nuts, and dietary fiber was notably insufficient. These findings underscore the need for targeted dietary interventions that promote fiber-rich plant foods, moderate excessive protein and refined carbohydrate intake to improve metabolic health in university populations.

## 6. Strengths and Limitations

The interviews were conducted by trained nutrition professionals who received specific instructions on the methodology and application of the Food Consumption Questionnaire (FCQ). Additionally, other dietary assessment tools, such as the 24-h recall, were utilized to ensure a rigorous and reliable data collection process. However, our study had some limitations. One key limitation was reliance on self-reported dietary data, which may lead to an underestimation of food intake. Furthermore, the sample size was relatively small, and the number of male participants was lower. While the sample was sufficient for statistical comparisons, the reduced size may have limited the statistical power for gender-specific analyses.

## Figures and Tables

**Table 1:** Sociodemographic characteristics of the participants.

	Female	Male	Total
Gender	106 (77.9%)	30 (22.1%)	136
Age (years) M ± SD	20.22 ± 1.95	20.76 ± 1.86	20.33 ± 1.94
BMI (kg/m^2^) M ± SD	22.32 ± 3.35	22.41 ± 3.68	22.38 ± 3.42
Family History			
Cancer F, (%)	4 (3.7%)	2 (6.6%)	6 (4.4%)
Cancer, and Hypertension F, (%)	3 (2.8%)	-	3 (2.2%)
Diabetes, F (%)	12 (11.3%)	3 (10%)	15 (11.0%)
Diabetes, Cancer, and Hypertension, F (%)	30 (28.30%)	2 (6.6%)	32 (23.5%)
Hypertension, F (%)	4 (3.7%)	2 (6.6%)	6 (4.4%)
Hormonal Disorders, F (%)	3 (2.83%)	-	3 (2.2%)
None, F (%)	50 (47.1%)	20 (66.6%)	70 (51.1%)
Personal history of illnesses			
Anemia, F (%)	6 (5.6%)	-	6 (4.4%)
Diabetes, F (%)	2 (1.8%)	-	2 (1.5%)
Hormonal Disorders, F (%)	11 (10.3%)	-	11 (8.1%)
None, F (%)	87 (82.0%)	29 (96.6%)	117 (86%)
Physical activity			
Low, F (%)	31 (29.2%)	9 (30%)	40 (29.4%)
Moderate, F (%)	40 (3.7%)	14 (46.6%)	54 (39.7%)
Intense, F (%)	13 (12.2%)	3 (10%)	17 (12.5%)
Very intense, F (%)	2 (1.8%)	2 (6.6%)	4 (2.9%)
Sedentary, F (%)	20 (18.6%)	1 (3.3%)	21(15.4%)
Smoking habit			
Smoker, F (%)	13 (12.2%)	8 (26.6%)	21 (15.4%)
Occasional-Smoker, F (%)	22 (20.7%)	7 (23.3%)	29 (21.3%)
Non-Smoker, F (%)	67 (63.2%)	11 (36.6%)	79 (58.1%)
Ex-Smoker, F (%)	4 (3.7%)	3 (10%)	7 (5.1%)
Alcohol consumption habits			
Weekends, F (%)	68 (64.1%)	16 (53.3%)	84 (61.8%)
3 or more times a week, F (%)	4 (3.7%)	6 (20%)	10 (7.4%)
None, F (%)	34 (32.0%)	7 (23.3%)	42 (30.9%)

Gender, age, and body mass index (BMI) data were expressed as the mean (M) ± standard deviation (SD). Sociodemographic and lifestyle data were expressed as frequencies (F) and percentages (%). Student’s *t*-test was used to compare means; a *p*-value of <0.05 was considered significant.

**Table 2:** Food consumption by food groups evaluated in the study population.

	Men	Women	
Consumption by Food Group (servings/day)	Mean ± SD	Mean ± SD	***p***-Value
Vegetables and greens, 4–5	4.01 ± 2.5	4.42 ± 2.35	0.11
Fruits, 2	3.84 ± 2.58	3.68 ± 1.92	0.67
Nuts, 1	0.82 ± 0.77	0.89 ± 0.88	0.76
Cereals and grains, 9–11 men/6–9 women	9.06 ± 4.37	8.43 ± 3.17	0.39
Legume, 2	1.75 ± 1.25	1.55 ± 0.86	0.37
Meat, 3–4	6.39 ± 2.14	5.13 ± 1.70	0.002 *
Dairy, 3	4.63 ± 2.20	3.87 ± 1.76	0.11
Beverages with added sugars, 0	3.04 ± 2.70	2.0 ± 2.16	0.01 *

Data were expressed as the mean ± standard deviation (SD). Student’s *t*-test was used to compare means; a *p*-value of <0.05 was considered significant. * Represents statistical differences between genders. Recommended portions according to the Ministry of Health, dietary guidelines for the Mexican population [19].

**Table 3:** Analysis of the diet in caloric energy and macronutrient distribution.

	Men	Woman	*p*-Value
	M ± SD	M ± SD	
Caloric energy, kcal/day	2278 ± 752	2008 ± 635	0.07
Carbohydrates, g/day	324 ± 120	292 ± 100	0.17
Proteins, g/day	218 ± 65	183 ± 56	0.01 *
Lipids, g/day	52 ± 16	43 ± 14	0.01 *
Fiber, g/day	19 ± 7.5	19 ± 6.6	0.92
Sugary drinks and sugar, g/day	30 ± 27	20 ± 22	0.04 *
Carbohydrates, (%)	56 ± 6	58 ± 5	0.33
Proteins, (%)	39 ± 8	37 ± 5	0.12
Lipids, (%)	21 ± 4	20 ± 3	0.13

Data were expressed as the mean (M) ± standard deviation (SD) for total caloric intake, macronutrients, and dietary fiber. Student’s *t*-test was used to compare means, and a *p*-value of <0.05 was considered statistically significant. * Represents statistical differences between genders.

**Table 4:** Adherence of the study population to different dietary patterns.

	Men	Women	Total
Ultra-processed dietary pattern, F (%)	16 (55.1)	30 (28.3)	46 (34.1)
Variety dietary pattern, F (%)	2 (6.8)	37 (34.9)	39 (28.9)
Traditional Mixed Mexican dietary pattern, F (%)	11 (37.9)	39 (36.8)	50 (37.0)

Data were expressed in frequencies (F) and percentages (%).

## Data Availability

The datasets generated during and/or analyzed during the current study are available from the corresponding author upon reasonable request due to ethical reasons.

## Supplementary Materials

The following supporting information can be downloaded at: https://www.mdpi.com/article/10.3390/nu18030532/s1, Table S1: Factor-loading matrix for three major dietary patterns identified by principal component analysis.

## References

[1] Maillet M.A., Grouzet F.M.E. (2023). Understanding changes in eating behavior during the transition to university from a self-determination theory perspective: A systematic review. J. Am. Coll. Health.

[2] Sprake E.F., Russell J.M., Cecil J.E., Cooper R.J., Grabowski P., Pourshahidi L.K., Barker M.E. (2018). Dietary patterns of university students in the UK: A cross-sectional study. Nutr. J..

[3] Bretti V., Yanine M.J., Marcos N. (2022). Relación Entre la Calidad de la Dieta, el Índice de Masa Corporal y el Perímetro de Cintura en Estudiantes de la Facultad de Ciencias de la Salud de la Universidad del Desarrollo, Concepción, 2022. Undergraduate Dissertation.

[4] Fernández F., González-Céspedes L. (2023). Calidad de la dieta y estado nutricional de un grupo de estudiantes de una universidad pública de Paraguay. Rev. Salud Publica Parag..

[5] Estrada Nava E.Y., Veytia López M., Pérez-Gallardo L., Guadarrama Guadarrama R., Gaona Valle L.S. (2020). Relación de la grasa corporal con la alimentación emocional y calidad de la dieta en universitarios de México. Arch. Latinoam. Nutr..

[6] Wagner M.C., Esquercia L.H., Ravelli S.D. (2021). Calidad de la dieta en estudiantes universitarios de la ciudad de Santa Fe. FABICIB.

[7] Vega S., León C., Tolentino R.G., Radilla M. (2019). Intervención para la incentivación del consumo de verduras y frutas como estrategia para la disminución del exceso de peso en adolescentes de la Ciudad de México. Rev. Esp. Nutr. Comunitaria.

[8] Campos-Nonato I., Galván-Valencia Ó., Hernández-Barrera L., Oviedo-Solís C., Barquera S. (2023). Prevalencia de obesidad y factores de riesgo asociados en adultos mexicanos: Resultados de la Ensanut 2022. Salud Pública México.

[9] Marrón-Ponce J.A., Flores M., Cediel G., Monteiro C.A., Batis C. (2019). Associations between consumption of ultra-processed foods and intake of nutrients related to chronic non-communicable diseases in Mexico. J. Acad. Nutr. Diet..

[10] Lazcano-Ponce E.C., Shamah-Levy T. (2023). La Salud de Los Mexicanos en Cifras: Resultados de la Ensanut 2022 [Internet]. https://www.saludpublica.mx/index.php/spm/article/view/14809.

[11] Ramírez-Toscano Y., Canto-Osorio F., Carnalla M., Colchero M.A., Reynales-Shigematsu L.M., Barrientos-Gutiérrez T., López-Olmedo N. (2023). Patrones de consumo de alcohol en adolescentes y adultos mexicanos: Ensanut Continua 2022. Salud Pública México.

[12] Diario Oficial de la Federación (2012). Norma Oficial Mexicana NOM-012-SSA3-2012, Que Establece Los Criterios Para la Ejecución de Proyectos de Investigación Para la Salud en Seres Humanos.

[13] Vázquez C., Escalante A., Huerta J., Villarreal M.E. (2021). Effects of the consumption frequency of ultra-processed foods and its association with nutritional status parameters on Mexican labor force population. Rev. Chil. Nutr..

[14] World Health Organization (2022). Obesity and Overweight. https://www.who.int/news-room/fact-sheets/detail/obesity-and-overweight.

[15] Yu J.J., Ye J. (2023). Resilience is associated with physical activity and sedentary behaviour recommendations attainment in Chinese university students. Complement. Ther. Clin. Pract..

[16] Ramírez-Silva I., Jiménez-Aguilar A., Valenzuela-Bravo D., Martínez-Tapia B., Rodríguez-Ramírez S., Gaona-Pineda E.B., Angulo-Estrada S., Shamah-Levy T. (2016). Methodology for estimating dietary data from the semi-quantitative food frequency questionnaire of the Mexican National Health and Nutrition Survey 2012. Salud Pública México.

[17] Lizaur A.B.P., Laborde L.M., González B.P. (2014). Sistema Mexicano de Alimentos Equivalentes.

[18] Osborne J., Osborne J.W., Costello A.B., Kellow J.T., Osborne J.W. (2008). Best practices in exploratory factor analysis. Best Practices in Quantitative Methods.

[19] Secretariat of Health (2023). Healthy and Sustainable Dietary Guidelines for the Mexican Population.

[20] World Health Organization (2018). Alimentación Sana. https://www.who.int/es/news-room/fact-sheets/detail/healthy-diet.

[21] Clark P., Mendoza-Gutiérrez C.F., Montiel-Ojeda D., Denova-Gutiérrez E., López-González D., Moreno-Altamirano L., Reyes A. (2021). A healthy diet is not more expensive than less healthy options: Cost-analysis of different dietary patterns in Mexican children and adolescents. Nutrients.

[22] Blondin S.A., Mueller M.P., Bakun P.J., Choumenkovitch S.F., Tucker K.L., Economos C.D. (2016). Cross-Sectional Associations between Empirically-Derived Dietary Patterns and Indicators of Disease Risk among University Students. Nutrients.

[23] Castro M.A., Baltar V.T., Marchioni D.M., Fisberg R.M. (2016). Examining Associations between Dietary Patterns and Metabolic CVD Risk Factors: A Novel Use of Structural Equation Modelling. Br. J. Nutr..

[24] Betancourt-Nuñez A., Márquez-Sandoval F., González-Zapata L.I., Babio N., Vizmanos B. (2018). Unhealthy dietary patterns among healthcare professionals and students in Mexico. BMC Public Health.

[25] Hu F.B., Rimm E.B., Stampfer M.J., Ascherio A., Spiegelman D., Willett W.C. (2000). Prospective study of major dietary patterns and risk of coronary heart disease in men. Am. J. Clin. Nutr..

[26] Denova-Gutiérrez E., Castañón S., Talavera J.O., Gallegos-Carrillo K., Flores M., Dosamantes-Carrasco D., Willett W.C., Salmerón J. (2010). Dietary patterns are associated with metabolic syndrome in an urban Mexican population. J. Nutr..

[27] Marrón-Ponce J.A., Sánchez-Pimienta T.G., Rodríguez-Ramírez S., Batis C., Cediel G. (2023). Ultra-processed foods consumption reduces dietary diversity and micronutrient intake in the Mexican population. J. Hum. Nutr. Diet..

[28] Oliveros E., Somers V.K., Sochor O., Goel K., Lopez-Jimenez F. (2014). The concept of normal weight obesity. Prog. Cardiovasc. Dis..

[29] Ballesteros-Torres J.M., Escalante-Aburto A., Villarreal-Arce M.E., Caballero-Prado C.J. (2024). Exploring the impact of protein supplement source on body composition in women practicing anaerobic resistance exercise: A pilot study. Nutrients.

[30] Plascencia R., Karam D. (2010). Comisión Nacional de los Derechos Humanos.

[31] Pereira-Santos M., da Mota Santana J., de Carvalho A.C.N., Freitas F. (2016). Dietary patterns among nutrition students at a public university in Brazil. Rev. Chil. Nutr..

[32] Almoraie N.M., Alothmani N.M., Alomari W.D., Al-Amoudi A.H. (2024). Addressing nutritional issues and eating behaviours among university students: A narrative review. Nutr. Res. Rev..

[33] Ünal G. (2024). Cooking and food skills and their relationship with adherence to the Mediterranean diet in young adults attending university: A cross-sectional study from Türkiye. Nutr. Bull..

[34] Antonopoulou M., Mantzorou M., Serdari A., Bonotis K., Vasios G., Pavlidou E., Trifonos C., Vadikolias K., Petridis D., Giaginis C. (2020). Evaluating Mediterranean diet adherence in university student populations: Does this dietary pattern affect students’ academic performance and mental health?. Int. J. Health Plan. Manag..

[35] Martínez-Rodríguez A., Vidal-Martínez L., Martínez-Olcina M., Miralles-Amorós L., Sánchez-Sáez J.A., Ramos-Campo D.J., Sánchez-Sánchez J., Martínez-Amorós N., Cheikh-Moussa K., Asencio-Mas N. (2023). Study the effect of an innovative educational program promoting healthy food habits on eating disorders, Mediterranean diet adherence and body composition in university students. Healthcare.

[36] Ünal G., Özenoğlu A. (2025). Association of Mediterranean diet with sleep quality, depression, anxiety, stress, and body mass index in university students: A cross-sectional study. Nutr. Health.

[37] Campos W.d., Stabelini Neto A., Bozza R., Ulbrich A.Z., Bertin R.L., Mascarenhas L.P.G., Silva SGda Sasaki J.E. (2010). Atividade física, consumo de lipídios e fatores de risco para aterosclerose em adolescentes. Arq. Bras. Cardiol..

[38] Duno M., Acosta E. (2019). Percepción de la imagen corporal en adolescentes universitarios. Rev. Chil. Nutr..

[39] Shan Z., Rehm C.D., Rogers G., Ruan M., Wang D.D., Hu F.B., Mozaffarian D., Zhang F.F., Bhupathiraju S.N. (2019). Trends in dietary carbohydrate, protein, and fat intake and diet quality among US adults, 1999–2016. JAMA.

[40] Whittaker J., Wu K. (2021). Low-fat diets and testosterone in men: A systematic review and meta-analysis of intervention studies. J. Steroid Biochem. Mol. Biol..

[41] Grabia M., Perkowski J., Socha K., Markiewicz-Żukowska R. (2024). Female athlete triad and relative energy deficiency in sport (REDs): Nutritional management. Nutrients.

[42] Escalante-Aburto A., Peña-Becerril M. Integration of cardiovascular risk assessment as a strategy for awareness and habit improvement during university studies. Proceedings of the 2025 Institute for the Future of Education Conference (IFE).

[43] Pivetta L., Borgatello C.I., Bove M.F., Bussy J.F. (2013). Evaluación de la ingesta de proteínas en jugadores de rugby de planteles superiores de clubes de Rosario (Argentina). Invenio.

[44] Martínez-Segura A., Cortés-Castell E., Rizo-Baeza M.M., Gil-Guillén V.F. (2015). Valoración de la dieta de usuarios de sala de musculación con dismorfia muscular (vigorexia). Nutr. Hosp..

[45] Cuenca-Sánchez M., Navas-Carrillo D., Orenes-Piñero E. (2015). Controversies surrounding high-protein diet intake: Satiating effect and kidney and bone health. Adv. Nutr..

[46] Zhang X., Sergin I., Evans T.D., Jeong S.-J., Rodriguez-Velez A., Kapoor D., Chen S., Song E., Holloway K.B., Crowley J.R. (2020). High-protein diets increase cardiovascular risk by activating macrophage mTOR to suppress mitophagy. Nat. Metab..

[47] World Health Organization (2025). A Healthy Lifestyle: WHO Recommendations. https://www.who.int/europe/news-room/fact-sheets/item/everyday-actions-for-better-health-who-recommendations.

[48] Togo P., Osler M., Sørensen T.I.A., Heitmann B.L. (2001). Food intake patterns and body mass index in observational studies. Int. J. Obes..

