# Supplementary material for: Dietary Trends and Lifestyle Habits Among University Students: Analysis of Consumption Patterns and Nutritional Risks
Source: Nutrients. 2026 Feb 5;18(3):532. doi: 10.3390/nu18030532 (PMC12899427; doi:10.3390/nu18030532)
Supplement: Supplementary file 1 [file nutrients-18-00532-s001.zip › nutrients-4103972-supplementary.pdf]

Table S1. Factor-loading matrix for three major dietary patterns identified by principal component analysis

	**Dietary patterns**		
	**Ultra-processed dietary pattern**	**Variety dietary pattern**	**Traditional Mixed Mexican dietary pattern**
Vegetables and greens	-.11	**.34**	**.47**
Fruit	-.22	**.53**	.27
Whole-wheat bread	.24	**.35**	.11
White bread and refined flours	**.62**	.01	.19
Sweet bread	.13	.10	.17
Red meats and processed meats	**.53**	-.01	**.43**
Chicken and fish	-.017	**.62**	**.42**
Chicken eggs	-.08	.29	**.43**
Legumes	.13	.10	**.54**
Natural dairy products	.24	**.53**	.23
Sugary dairy products	**.47**	.02	.04
Vegetable drinks (almond and soy)	.10	**.57**	-.20
Vegetable Oils (soybean, corn, and canola)	**.46**	.17	-.04
Olive oil	-.04	**.61**	.14
Sugary drinks	**.50**	-.20	.12
American coffee and coffee with milk	-.13	.12	**.57**
Beer	.03	-.24	-.19
Fried foods	**.75**	-.18	-.18
Commercial pastries and desserts	**.38**	.01	-.05
Mexican food	**.61**	-.14	.13
Fast food	**.58**	-.04	.02
